# [Retracted] Restoration of the tumor-suppressor function to mutant p53 by *Ganoderma lucidum* polysaccharides in colorectal cancer cells

**DOI:** 10.3892/or.2026.9181

**Published:** 2026-08-17

**Authors:** Dan Jiang, Lingyao Wang, Tong Zhao, Zhaoyu Zhang, Renxia Zhang, Jingji Jin, Yong Cai, Fei Wang

Oncol Rep 37: 594–600, 2017; DOI: 10.3892/or.2016.5246

Following the publication of the above paper, it was drawn to the Editor's attention by a concerned reader that western blots portraying the protein p53 in Figs. 1C and 4C had previously appeared as p53 bands in an article published in 2010 in the journal *PLoS One* that featured the author Fei Wang in common, although the exprimental conditions were reported to be different in the previous paper. In addition, regarding the flow cytometric plots shown in Fig. 1D on p. 596, the ‘HCT116 P53^’ plots for the NTC (upper row) and 5-FU (lower row) experiments appeared to show similar groupings of dots in specific areas of the quadrants, which would not have been anticipated if these experiments had been performed discretely under different experimental conditions, suggesting a fundamental flaw either in the way in which these experiments were performed or in how the results were outputted.

Given that these issues have come to light, the Editor of *Oncology Reports* has decided that this paper should be retracted from the Journal on account of a lack of confidence in the presented data. After contacting the authors, they accepted the decision to retract the paper. The Editor apologizes to the readership for any inconvenience caused.

